# Perceiver CPI: a nested cross-attention network for compound–protein interaction prediction

**DOI:** 10.1093/bioinformatics/btac731

**Published:** 2022-11-23

**Authors:** Ngoc-Quang Nguyen, Gwanghoon Jang, Hajung Kim, Jaewoo Kang

**Affiliations:** Department of Computer Science and Engineering, Korea University, Seoul 02841, Republic of Korea; Department of Computer Science and Engineering, Korea University, Seoul 02841, Republic of Korea; Interdisciplinary Graduate Program in Bioinformatics, Korea University, Seoul 02841, Republic of Korea; Department of Computer Science and Engineering, Korea University, Seoul 02841, Republic of Korea; Interdisciplinary Graduate Program in Bioinformatics, Korea University, Seoul 02841, Republic of Korea; AIGEN Sciences, Seoul 04778, Republic of Korea

## Abstract

**Motivation:**

Compound–protein interaction (CPI) plays an essential role in drug discovery and is performed via expensive molecular docking simulations. Many artificial intelligence-based approaches have been proposed in this regard. Recently, two types of models have accomplished promising results in exploiting molecular information: graph convolutional neural networks that construct a learned molecular representation from a graph structure (atoms and bonds), and neural networks that can be applied to compute on descriptors or fingerprints of molecules. However, the superiority of one method over the other is yet to be determined. Modern studies have endeavored to aggregate information that is extracted from compounds and proteins to form the CPI task. Nonetheless, these approaches have used a simple concatenation to combine them, which cannot fully capture the interaction between such information.

**Results:**

We propose the Perceiver CPI network, which adopts a cross-attention mechanism to improve the learning ability of the representation of drug and target interactions and exploits the rich information obtained from extended-connectivity fingerprints to improve the performance. We evaluated Perceiver CPI on three main datasets, Davis, KIBA and Metz, to compare the performance of our proposed model with that of state-of-the-art methods. The proposed method achieved satisfactory performance and exhibited significant improvements over previous approaches in all experiments.

**Availability and implementation:**

Perceiver CPI is available at https://github.com/dmis-lab/PerceiverCPI.

**Supplementary information:**

Supplementary data are available at *Bioinformatics* online.

## 1 Introduction

Drug development is a high-cost low-efficient process. New drug approval typically requires 10–15 years and costs 2.8 billion dollars on an average (Wouters *et al.*, 2020). Various approaches based on artificial intelligence have been introduced to alleviate this problem. In recent years, traditional machine learning (ML) algorithms have been deployed to model the prediction of the interaction between compounds and proteins as a binary classification problem (Bahi and Batouche, 2021). However, binding affinity, which indicates the interaction strength of the drug–target pair, is a continuum value; hence, considering compound–protein interaction (CPI) as a regression problem is both effective and sufficient.

Through the binding mechanism, drugs can have a positive or negative influence on the functions carried out by proteins, which affect the targeted disease conditions (You *et al.*, 2018). Understanding drug–target binding affinity makes it possible to identify candidate drugs that can inhibit or stimulate a given protein. Researchers have attempted to exploit meaningful information from given proteins and compounds. Notably, in terms of protein information extraction, most previous approaches consider the protein sequence as a plain text and then use a 1D convolutional neural network (1DCNN) with different methods of protein sequence numbering. Nevertheless, two types of models have shown excellent performance in terms of obtaining information from chemical compounds: deep neural networks (DNNs) such as a multiple layer perceptron (MLP) neural network, and 1DCNN performing on descriptors or fingerprints, and graph neural networks (GNNs) and their variants for extracting knowledge from a graph-structured dataset (Yang *et al.*, 2019).

With respect to the first approach for molecular descriptors, Öztürk *et al.* (2018) proposed DeepDTA, which adopts two 1DCNNs to perform on raw sequences and the simplified molecular-input line-entry system (SMILES) (Weininger, 1988) as one-hot vectors. Using 1DCNN, the authors aimed to extract local residues and atomic features to predict binding affinity. DeepConv-DTI (Lee *et al.*, 2019) followed a similar idea of DeepDTA by introducing a deep learning (DL) model to predict CPIs using raw protein sequences with Morgan/circular fingerprints (Morgan, 1965) as a compound representation. They used a 1DCNN on entire sequences of proteins to capture local residue patterns, while applying MLP neural network on molecular fingerprints to get drug features. Subsequently, Lee *et al.* concatenated aforementioned features, then transmitted them to a fully connected layer and predicted the property.

Regarding the second method, GNNs that follow a neighborhood aggregation scheme have become increasingly popular for graph-structured data (Scarselli *et al.*, 2009). Numerous variants of GNN models have been proposed to achieve state-of-the-art (SOTA) performances in graph-based tasks in various fields of deep learning. Aware of the strength of GNNs, Nguyen *et al.* (2021) converted a compound representation into a graph represented by nodes (atoms) and edges (bonds); they, then, used four types of GNNs, graph convolutional networks (GCNs), graph attention networks (GATs), graph isomorphism networks (GINs) and a combination of GCNs and GATs, to capture molecular information. The knowledge extracted from atoms and bonds was then concatenated with the output of three 1DCNNs, which were used to learn different levels of abstract features from raw protein sequences.

Transformers (Vaswani *et al.*, 2017) have shown a good performance in many AI fields, such as computer vision and natural language processing. Inspired by their potential to capture features between two sequences, Chen *et al.* (2020) proposed TransformerCPI, which is based on the architecture of an autoregressive encoder–decoder, using a combination of multiheaded attention and positional feed-forward to perform the CPI task. In their approach, molecular graphs were propagated to a *GCN* to obtain atomic features. Meanwhile, protein sequences were converted into sequential representations by separating a protein sequence into an overlapping 3-g amino acid sequence. Then, all words were translated into real-valued embeddings using the pretraining approach. The output was processed through 1DCNNs to obtain the final representation of the protein. Subsequently, these two representations were combined using a modified self-attention mechanism followed by MLPs. Motivated by the effectiveness of the self-attention mechanism, HyperattentionDTI was created (Zhao *et al.*, 2022). The model was designed to input both compounds and proteins as plain sequences to two stacked 1DCNNs. In contrast to previous attention-based models, HyperattentionDTI inferred an attention vector by using a Sigmoid activation function rather than using a Softmax activation function.

The drawbacks of the existing approaches can be summarized as follows:

1. Because molecular descriptor vectors or fingerprints [such as extended-connectivity fingerprint (ECFP)] contain useful chemical knowledge from the start, the use of molecular fingerprints and molecular descriptors might lead to a better performance than using complex graphs on small datasets. However, owing to the representation’s simplification, models deploying them may underfit larger datasets.
2. On the other hand, GNNs must always learn a meaningful chemical space embedding from scratch. In addition, because of the global pooling step, which is simply chosen as the sum or average of all atomic features, over-smoothing and information loss are also crucial issues for GNNs.
3. Integration of the compound network’s and protein network’s representation is often performed by a simple concatenation, which is practically unsuitable for revealing the relationship between these molecules in practice.
4. Obtaining informative messages from protein sequences is a focus of research not only in CPI tasks but also in the general bioinformatics. Most current approaches consider protein sequences as plain texts, which cannot sufficiently reveal the real 3D structures of proteins.

In this study, we developed Perceiver CPI, a deep-learning model that addresses three of the abovementioned challenges (1, 2 and 3). Our approach is mainly inspired by that of Perceiver IO (Jaegle *et al.*, 2021b) and a directed message-passing neural network (D-MPNN) (Yang *et al.*, 2019). The contributions of this study are summarized as follows:

- To avoid over-smoothing and information loss problems, we propose a novel method to enrich the representation of compounds by combining the information from both ECFPs and graph information.
- To the best of our knowledge, Perceiver CPI is the first approach to use nested cross-attention for capturing the relations between protein and molecule representations.
- Experimental results show that Perceiver CPI can achieve SOTA performance in novel pair and novel compound settings, and is competitive or slightly better than the baseline models in a novel protein setting.

## 2 Materials and methods

### 2.1 Feature encoding

#### 2.1.1 Compound information encoding

Unlike previous approaches, which have commonly used either ECFP or molecular graph information constructed from SMILES, our proposed approach adopts ECFP to enrich the information extracted from the compound using D-MPNN (Yang *et al.*, 2019). More specifically, we represent a molecule *s* using two forms:

- A Morgan/circular fingerprint vector *M_s_* as a binary vector, which indicates the existence of specific substructures. The Morgan algorithm searches each atom of the molecule and obtains all possible paths through the atom with a specific radius. Then, each unique path is hashed into a number based on a maximum of bit number.
- A molecular graph $G_{s}(A,B)$, which represents the interactions between a set of atoms *A* by a set of bonds *B*.

We then process *M_s_* and *G_s_* through a MLP neural network and D-MPNN, respectively. Owing to its ability to approximate any continuous mapping, the MLP neural network is used to capture complex non-linear relationship features from *M_s_* to yield *O_Ms_* as the output. D-MPNN operates on hidden states $h_{vw}^{t}$ and messages $m_{vw}^{t}$ associated with directed edges (bonds) instead of messages associated with vertices (atoms). Each bond in the graph has a hidden state (i.e. feature vector) that contains atomic features (atomic number, number of bonds for each atom, formal charge, chirality, number of bonded hydrogens, hybridization, aromaticity and atomic mass) and bond features [bond type (single/double/triple/aromatic), conjugation, ring membership and stereochemistry] (Stokes *et al.*, 2020). For each bond *B_vw_*, we aggregate the function of the hidden states of all arriving neighboring bonds with the hidden state $h_{vw}^{t}$ itself. Then, the hidden state of edge $h_{vw}^{t}$ is updated using the obtained message and the previous hidden state of the atom. In other words, the hidden state of bond *B_vw_* is obtained by updating the old hidden state with the newly obtained message. The corresponding message-passing update equations from atom *A_v_* to atom *A_w_* are as follows:

(1) $$\{\begin{array}{cc} m_{vw}^{t+1}=\sum_{k\in \left\{N\left(v\right)\setminus w\right\}}Av\mathrm{erage}(x_{v},x_{k},h_{kv}^{t}) & \\ h_{vw}^{t+1}=f_{t}(h_{vw}^{t},m_{vw}^{t+1}) & \end{array}$$

where *x* is the feature of the corresponding atom *A*, *f_t_* is a MLP layer.

Specifically, in the message-passing phase, all messages arriving at bond *B_vw_* are aggregated using a permutation-invariant aggregation function *Average*. Rather than using the summation function as suggested by the original D-MPNN, which caused model instability when training on small datasets, we adopt the average function *Average* to help the model update gradually. The aggregated representation is then combined with the existing hidden state via the MLP *f_t_*, resulting in an updated node feature vector *h_vw_*. Notably, all hidden states are initially set to $h_{vw}^{0}=\varphi (f(\mathrm{concat}(x_{v},e_{vw})))$ (with *e_vw_* as the feature of bond *B_vw_*, φ is ReLU activation function). The main idea behind the message-passing technique is to prevent the distortion of messages between atoms. For example, the message from *A_n_* to $A_{n+1}$ will only be propagated to $A_{n+2}$ and $A_{n+3}$ in the next iteration, whereas, in a conventional MPNN, it will be sent to node *A_n_*, creating an unnecessary loop in the message-passing process. In the readout phase, we use one more average function to construct a final representation *O_Gs_*.

**Fig. 1.**
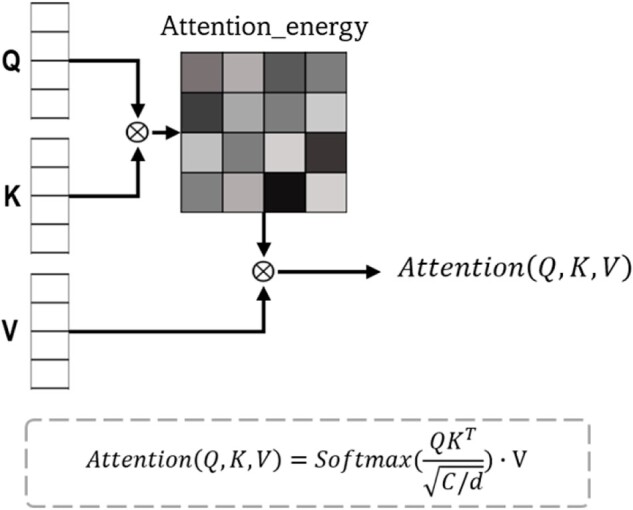
Demonstration of attention blocks. While the self-attention block accepts inputs from only single source, cross-attention blocks receive information from two sources

Finally, after having two outputs *O_Ms_* from MLP net and *O_Gs_* from D-MPNN, we combine this information by adopting a cross-attention mechanism:

(2) $$Q=f_{Q}(O_{Ms});K=f_{K}(O_{Gs});V=f_{V}(O_{Gs})$$

(3) $$\mathrm{Attention}\_\mathrm{energy}=\mathrm{Softmax}\left(\frac{QK^{T}}{\sqrt{C/d}}\right)$$

(4) $$\mathrm{Com}p_{s}=\mathrm{CrossAttention}(Q,K,V)=\mathrm{Attention}\_\mathrm{energy}*V$$

where *Q* is created from the output of the Morgan fingerprint MLP *O_Ms_*, and *K* and *V* are generated from the output *O_Gs_* of the D-MPNN by the projection functions $f=w^{T}x+b$ (where w and *b* are weight and bias, respectively). *C* and *d* are the embedding dimensions and number of heads, respectively. Figure 1 visualizes the attention module. Note that, *O_Ms_* and *O_Gs_* are 1D arrays. In our experiments, one more projection function *f*_0_ was used for a dimension reduction purpose at the end of the block. We found that a single-head cross-attention outperformed other multi-head cross-attentions.

Using two modalities, we provide multiple views from compounds to the model; hence, Perceiver CPI is able to learn comprehensive patterns precisely. The ECFP provides information on the existence of substructures, whereas the graph representation considers the carrying knowledge that shows how they connect to one another.

#### 2.1.2 Protein information encoding

The protein *t* was encoded using the tasks assessing protein embeddings (TAPE) tokenizer, where the initial feature of each residue was represented by the corresponding number following the UniRep Vocabulary (Rao *et al.*, 2019). We used this one-hot encoding scheme for protein sequences, mainly because it is the simplest method to construct a unified representation (UniRep), which is broadly applicable and generalized to unseen regions of sequence space (Alley *et al.*, 2019). The input is zero-padded to ensure that the number of output features remains fixed and then propagated into the blocks of 1DCNNs. Finally, we obtain the final output features. To help the model learn more deeply, we use the skip connection type to gradually change the weight of the network (He *et al.*, 2016). Skip connections suggest skipping some of the layers in the neural network and feeding the output of one layer as the input to the next layers, thereby ensuring feature reusability to avoid the shattered gradient problem. The shattered gradient problem occurs in DNNs when the gradients resemble white noise and negatively impact the training (Balduzzi *et al.*, 2017). Residual connection resolves this by introducing a spatial structure to the gradients, thus stabilizing the training process. Eventually, the output of the 1D convolution block can be expressed as follows:

Algorithm 1 An algorithm for residual block of 1DCNN

**Require:** *M*, $emb_{in}\;\leftarrow \;\text{Conv}1D(emb_{0}),\lambda$

** Result:** $\mathrm{Pro}t_{t}\leftarrow emb_{\mathrm{out}}$

**for** *M* **do**

** **

$emb_{\mathrm{out}}\leftarrow \text{LN}$ ($\text{Conv}1D(emb_{in})$ + *emb_in_* * *λ*);

 $emb_{in}\leftarrow \text{GLU}(emb_{\mathrm{out}})$;

**end for**

where *M* is the number of 1DCNN layers, **_*λ*_** is fixed to isolate the effect of scaling and LN is a layer normalization function. *emb* represents the protein embedding with initialization *emb*_0_. Motivated by transformers, a combination of normalization and skip connection is observed to be helpful in facilitating the model’s capacity to learn of the model to protein information. Furthermore, the use of LN is intended to normalize the distributions of intermediate layers that might mitigate the gradient malformation to enable smoother gradients, faster training and better generalization accuracy. In particular, we use gated linear unit activation, a finite context approach through stacked convolutions, which can efficiently extract information from a sequence because it allows parallelization over sequential token features (Dauphin *et al.*, 2017).

### 2.2 Compound–protein interaction

After obtaining two output representations from the three-element networks, we need to precisely integrate them to ultimately teach the model to capture valuable information that reveals CPI properties. The effective fusion of these multiple input sources is becoming increasingly important, as these multi-modality features have been shown to generate highly accurate performances in various tasks. A significant fusion method synergistically combines the two modalities and guarantees that the resultant product reflects the binding features of the input modalities (Mohla *et al.*, 2020; Chen *et al.*, 2021). Inspired by Perceiver IO and Perceiver (Jaegle *et al.*, 2021a,b), we propose a novel method that leverages a highly asymmetric attention mechanism to distill compound information iteratively and then structure the final interaction representation using the protein information from a single cross-attention module. In the cross-attention block, we aim to force the model to capture patterns that show the effect of information from the compound on the protein information. In other words, we use this method because we primarily intend to determine how the protein reacts with the compound. More characteristically, after representing a compound, we process the compound latents by applying a series of self-attention modules to refine the compound representation. Finally, we combine *Comp_s_* and *Prot_t_* by applying a cross-attention module that maps latent arrays to the protein representation. The final interaction representation can be expressed as follows:

(5) $$Q=f_{Q}(\mathrm{Com}p_{s});K=f_{K}(\mathrm{Pro}t_{t});V=f_{V}(\mathrm{Pro}t_{t})$$

(6) $$\mathrm{Interactio}n_{s,t}=\mathrm{CrossAttention}(Q,K,V)$$

Using the cross-attention mechanism, we can model the semantic relevance between the protein and compound features, thus drawing attention to significant interaction information and benefiting the binding affinity prediction task. The cross-attention module generates cross-attention energy (also known as an attention map), which is then used to weight the feature map to achieve informative and discriminative feature representation. Moreover, the computation and memory complexity of generating attention energy in cross-attention are linear rather than quadratic, making the entire process more efficient.

### 2.3 Loss function and optimizer

In our experiment, we used the mean squared error (MSE) loss function with LAMB optimizer, which stands for ‘layer-wise adaptive moments optimizer for batch training’ (You *et al.*, 2019). As can be seen, the training may become unstable if this ratio is too high. However, the weights do not change rapidly enough if the ratio is too small. Using the trust ratio, LAMB enables the model to be more confident in each step and scale much larger batch sizes without causing divergence. The hyperparameters in our neural network are searched using Bayesian optimization algorithms.

### 2.4 Benchmark datasets

To compare SOTA models with the proposed Perceiver CPI model and analyze its performance, we used three well-known benchmark datasets. To make use of the complementary information captured by the various bioactivity types, including dissociation constant (*K_d_*), inhibition constant (*K_i_*) or the half maximal inhibitory concentration (*IC*_50_), Tang *et al.* (2014) introduced a model-based integration approach called KIBA to generate an integrated drug–target bioactivity matrix. KIBA scores were created to optimize the consistency of the three measurements. The Davis dataset contains the interactions of 68 kinase inhibitors with 442 kinases covering >80% of the human catalytic protein kinome without missing interactions (Davis *et al.*, 2011). The original unit of the dataset is *K_d_* values; however, normalization of the label helps improve the performance. Hence, log transformation was applied to scale the label in the smaller range *pK_d_* = $-log_{10}(K_{d}/1e9)$. Specifically, we used KIBA and Davis from the open-source software named DeepPurpose (Huang *et al.*, 2021). For Metz data, Metz *et al.* (2011) presented a critical statistical analysis of kinomics screening data across 170 different protein kinases and establishing rigorous criteria. The PDBbind dataset contained 16 151 interactions (Wang *et al.*, 2005). After filtering and processing to qualify the dataset, 6689 unique pairs were retained (Li *et al.*, 2020). Table 1 shows the summary statistics for all datasets.

**Table 1.** Statistics of the benchmark datasets

Dataset	Proteins	Drugs	Interactions	Density (%)
Davis (Davis *et al.*, 2011)	442	68	30 056	100
KIBA (Tang *et al.*, 2014)	229	2068	117 657	24.84
Metz (Metz *et al.*, 2011)	170	1423	35 259	14.57
PDBbind (Wang *et al.*, 2005)	2079	5535	6989	0.06

**Fig. 2.**
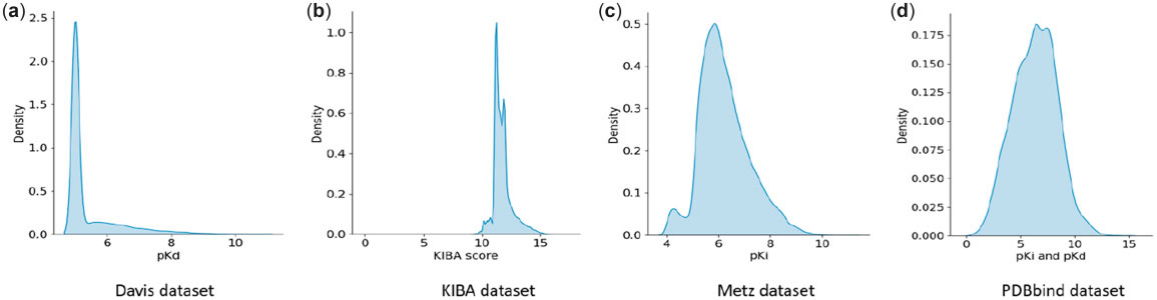
Visualization of benchmark datasets with kernel density estimation

Furthermore, the density of all four datasets is shown in Figure 2. We employed kernel density estimation, a fundamental data smoothing problem where inferences about the population are made based on a finite data sample to reveal the dataset density. Figure 2 indicates that almost all the Davis dataset binding affinity values were highly concentrated around five. In particular, 69.64% of the Davis dataset had affinity binding values of five, whereas 71.96% of the KIBA dataset were in the range from 11.1 to 12. Due to the skew distribution of Davis dataset and KIBA dataset, we empirically forced the model to perform a larger weight update for data points, which did not belong to the high density area 10 times larger than the others. For instance, in Davis dataset, the data points have binding affinity in the range from 0 to 5 were discounted by 0.5, while the out-ranged data points multiplied by 5. Conversely, the Metz and PDBbind datasets exhibited well-balanced distributions with fewer outliers than the others; however, the sparsity of these datasets is extremely high.

For a fair comparison, we also used the GPCR classification dataset to evaluate the enrichment factor (EF) from the Directory of Useful Decoys-Enhanced database (DUD-E) database. EF is used to show the performance of the model in finding true positives throughout the background database compared to random selection (Huang *et al.*, 2006). Moreover, EF reveals the concentration of the annotated ligands among the top-scoring compounds compared to their concentrations throughout the entire dataset. For instance, the enrichment factor at 1% is the percentage of ligands found when 1% of decoys were found (Jain, 2008). Decoys from the DUD-E database were extracted from the ZINC database and were commercially available compounds for virtual screening (Mysinger *et al.*, 2012). The 2D-similarity between the active compounds and decoy compounds are measured by calculating the Tanimoto distance. The statistics of the GPCR dataset are shown in Table 2 for training obtained from TransformerCPI, which was extracted from the GLASS database (Chan *et al.*, 2015). The dataset provides experimentally validated GPCR–ligand associations. A threshold of 6.0 was set to divide the original dataset into positive and negative sets. Finally, Table 3 presents the test sets collected from the GPCR and Diverse subsets in the DUD-E database.

**Table 2.** Statistic of GPCR dataset

Proteins	Compounds	Positive pairs	Negative pairs	Density (%)
356	5359	7989	7354	0.8

**Table 3.** Statistics of GPCR and diverse subsets from DUD-E database

Subset	Number of target	Actives	Decoys
GPCR subset	5	1480	99 856
Diverse subset	7	1759	107 591

## 3 Experimental results and discussion

### 3.1 Experimental results

**Model conversion:** As mentioned above, we considered the CPI task as a regression problem. Nevertheless, only a few methods have used a similar concept; we transformed binary classification models, such as TransformerCPI, DeepconvDTI and HyperattentionDTI into regression models by modifying their final layers. To maintain the original performance, the output of the last layer was transformed to a single perceptron node, and the loss function was altered to the MSE loss function.

**Experimental procedure:** Owing to the density of the Davis dataset and because the dataset approximately covers 80% of the human catalytic protein kinomes, we decided to perform three experiments: novel pair setting, novel compound setting and novel protein setting; only novel pair setting was applied on the KIBA and Metz datasets. Finally, the PDBbind dataset and GPCR dataset with the GPCR and the Diverse subsets from the DUD-E database were used for an additional analysis. To calculate the similarities, the protein similarity is measured by the percentage of the number of aligned amino acids over the total length (in Perceiver CPI, the length of the proteins was fixed to 500). Meanwhile, the compound similarity was calculated using the Tanimoto similarity function.

- Novel pair (Davis, KIBA and Metz): There were no overlaps between the training and test datasets. Neither the training compound nor the training protein appeared in the test set.
- Novel-hard pair (Davis): There were no overlaps between the training and test datasets. The testing interactions were highly selective for similarities less than 0.3 by comparing to training interactions.
- Novel compound (Davis): There were no intersections of compounds in the training set and compounds in the test set.
- Novel protein (Davis): There were no intersections of proteins in the training set and proteins in the test set.
- Cross-domain experiment (Davis and PDBbind): There were no overlaps between the training and test datasets. We trained the model with the Davis dataset and tested it with the PDBbind dataset.
- Enrichment factor analysis *[*GPCR, GPCR subset (DUD-E dataset), Diverse subset (DUD-E dataset)]: There were no overlaps between the training and test datasets. We trained the model with the GPCR dataset and tested it with subsets from the DUD-E dataset (the duplicated target ‘CXCR4’ was removed from the Diverse subset).

**Evaluation metric:** To evaluate the performance on the regression task, we used the mean squared error (MSE) metric to measure the performance of the models and the concordance index (CI) metric to evaluate the proportion of concordant prediction pairs per the total number of label pairs, which tells us whether the predicted binding affinity values of two random drug–target pairs were predicted in the same order as their truth values. In the enrichment factor analysis, we adopted an EF score at fraction 1% (EF${}_{1\%}$) to show the performance of the models in determining the annotated ligands among the top binding affinity compounds and the Boltzmann-enhanced discrimination of the receiver operating characteristic score to focus more on early enrichment with *α *= 80.5 (BEDROC${}_{\alpha =80.5}$).

**Cross-validation:** We applied five-fold cross-validation to calculate the performance of baseline models and Perceiver CPI in four experiments: novel pair, novel-hard pair, novel compound, novel protein. The validation set was taken arbitrarily from the training set following the ratio training: validation = 80%: 20% for all experiments.

Table 4 compares the performance of Perceiver CPI with five SOTA deep-learning baseline models for the three types of separations. Regarding the two principal tasks (novel pair and novel compound), Perceiver CPI showed remarkable performances. With reference to the novel pair setting, our proposed model achieved an MSE of 0.463(±0.013) and CI of 0.638(±0.028), whereas the competitors performed poorly. In the novel compound experiment, Perceiver CPI reached the lowest MSE (0.378(±0.010)) and had the highest value in CI (0.726(±0.017)). We discovered no significant difference between the previous approaches and our model in the novel protein tasks. Nonetheless, Perceiver CPI performed better than the others in terms of MSE and was competitive with the first-placed model in terms of CI metric. As shown in Table 5, in the most challenging setting, when the test set was significantly different from what the model trained on, the proposed method outperformed baseline compactors by providing precise predictions, resulting in the lowest MSE. In practice, the number of proteins is finite, and most of them will eventually be annotated, which means that the CPI task is mainly about finding a new compound with existing proteins in the real world. Interestingly, our model was also more stable than the others as indicated by its lower standard deviation among the validations.

**Table 4.** Comparison of the proposed method with SOTA model in terms of three settings from the Davis dataset with 5-fold cross-validation

Model	Novel pair		Novel compound		Novel protein	
	MSE	CI	MSE	CI	MSE	CI
DeepDTA (Öztürk *et al.*, 2018)	0.631(±0.059)	0.533(±0.027)	0.482(±0.034)	0.613(±0.029)	0.701(±0.045)	**0.759(±0.015)**
DeepConvDTI (Lee *et al.*, 2019)	0.598(±0.057)	0.546(±0.043)	0.512(±0.046)	0.681(±0.012)	0.789(±0.109)	0.714(±0.034)
TransformerCPI (Chen *et al.*, 2020)	0.549(±0.038)	0.490(±0.032)	0.522(±0.027)	0.592(±0.026)	0.708(±0.032)	0.676(±0.005)
GraphDTA (GINs) (Nguyen *et al.*, 2021)	0.846(±0.058)	0.459(±0.032)	0.452(±0.051)	0.670(±0.018)	0.970(±0.061)	0.660(±0.016)
HyperattentionDTI (Zhao *et al.*, 2022)	0.671(±0.045)	0.517(±0.013)	0.506(±0.015)	0.578(±0.019)	0.784(±0.063)	0.674(±0.020)
Perceiver CPI (ours)	**0.463(±0.013)**	**0.638(±0.028)**	**0.378(±0.010)**	**0.726(±0.017)**	**0.667(±0.018)**	0.758(±0.010)

*Note*: The metrics are MSE (the lower, the better) and CI (the higher, the better) (± standard deviation)

**Table 5.** Comparison of Perceiver CPI and other SOTA competitors on novel-hard pair setting

Model	MSE	CI
DeepDTA (Öztürk *et al.*, 2018)	0.948(±0.218)	0.565(±0.040)
DeepConvDTI (Lee *et al.*, 2019)	0.768(±0.290)	0.571(±0.052)
TransformerCPI (Chen *et al.*, 2020)	0.806(±0.254)	0.508(±0.071)
GraphDTA (GINs) (Nguyen *et al.*, 2021)	0.931(±0.314)	0.542(±0.070)
HyperattentionDTI (Zhao *et al.*, 2022)	0.873(±0.246)	0.600(±0.049)
Perceiver CPI (ours)	**0.701(±0.244)**	**0.609(±0.072)**

Considering the most challenging setting, the novel pair split settings with KIBA and Metz datasets, as shown in Table 6, DeepConvDTI achieved inferior performance using ECFP representation for compounds. Although ECFP captures useful information for CPI prediction, owing to its simplicity, the knowledge from compounds is still not fully used. Therefore, instead of using the ECFP independently, a combination of ECFP and graph representations of the compound was utilized to further improve performance. The two datasets contained many missing interactions, resulting in the underperformance of all models. In the KIBA dataset, Perceiver CPI attained a lower MSE than the baseline by 0.028 and a higher CI. In particular, it was extremely difficult to obtain correct predictions using the Metz dataset, which has a 14.57% density.

**Table 6.** Comparison of Perceiver CPI performance to SOTA baseline models in novel pair task from on KIBA and Metz datasets

Model	KIBA		Metz	
	MSE	CI	MSE	CI
DeepDTA (Öztürk *et al.*, 2018)	0.668(±0.055)	0.600(±0.011)	0.781(±0.060)	0.627(±0.011)
DeepConvDTI (Lee *et al.*, 2019)	0.550(±0.009)	0.635(±0.007)	0.703(±0.027)	0.671(±0.016)
TransformerCPI (Chen *et al.*, 2020)	0.630(±0.057)	0.563(±0.014)	1.081(±0.125)	0.557(±0.016)
GraphDTA (GINs) (Nguyen *et al.*, 2021)	0.698(±0.042)	0.591(±0.013)	1.232(±0.094)	0.615(±0.010)
HyperattentionDTI (Zhao *et al.*, 2022)	1.022(±0.062)	0.590(±0.015)	1.064(±0.080)	0.630(±0.013)
Perceiver CPI (ours)	**0.522(±0.010)**	**0.638(±0.013)**	**0.658(±0.016)**	**0.675(±0.012)**

Moreover, we performed a cross-domain experiment to determine the adaptability of our method to an unseen domain dataset. We chose two datasets (Davis and PDBbind) owing to their overlapping properties and measurements. First, we eliminated all overlapping interactions from the PDBbind dataset to the Davis dataset. Second, while we divided the Davis dataset into training and validation sets at a ratio of 80%:20%, the processed PDBbind dataset was used as a test set. The results in Table 7 show that Perceiver CPI significantly outperformed the baselines. The proposed approach achieved a higher performance on CI metrics than SOTA models, while exhibited a lower MSE. In other words, Perceiver CPI provides more precise predictions than the compared models. In particular, all models, including ours, performed poorly in the cross-domain experiment because of the quantity and quality of the training dataset.

**Table 7.** Results of the cross-domain experiment (trained on Davis and tested on PDBbind)

Model	MSE	CI
DeepDTA (Öztürk *et al.*, 2018)	4.716	0.500
DeepConvDTI (Lee *et al.*, 2019)	5.400	0.477
TransformerCPI (Chen *et al.*, 2020)	4.962	0.497
GraphDTA (GINs) (Nguyen *et al.*, 2021)	6.323	0.516
HyperattentionCPI (Zhao *et al.*, 2022)	5.946	0.410
Perceiver CPI (ours)	**4.612**	**0.532**

We tested the model and other classifiers and five docking-based programs [Gold (Jones *et al.*, 1997), Glide (Friesner *et al.*, 2004), Surflex (Jain, 2003), FlexX (Rarey *et al.*, 1996) and Blaster (Irwin *et al.*, 2009)] on subsets from the DUD-E database. We converted Perceiver CPI architecture into a classifier by changing the loss function from MSELoss to CrossEntropyLoss, as well as by transforming the last layer into a sigmoid function. Ligand enrichment among top-ranking hits is an important criterion for molecular docking and drug–target interactions. Table 8 reveals that Perceiver CPI achieved a better performance for multiple targets in an EF${}_{1\%}$ and BEDROC${}_{\alpha =80.5}$ than the other deep-learning models. However, the docking-based method outperformed the data-driven method for most protein targets on both metrics (Supplementary Table S7). Perhaps the combination of the two good methods might lead to an excellent performance. Moreover, the accumulation of extended datasets may enhance the predictions of the ML/DL models.

**Table 8.** Enrichment factor analysis results for subsets in the DUD-E database (UP: EF${}_{1\%}$, DOWN: BEDROC${}_{\alpha =80.5}$)

Family	DeepConvDTI	TransformerCPI	HyperattentionDTI	Perceiver CPI (ours)	Gold	Glide	Surflex	FlexX	Blaster
GPCR (DUD-E)	9.728(±11.534)	0.814(±1.178)	3.982(±3.119)	**16.366(±15.921)**	N/a	N/a	N/a	N/a	11.8(±8.136)
(DUD-E)	0.152(±0.174)	0.018(±0.040)	0.071(±0.058)	0.236(±0.177)	**0.282(±0.154)**	0.198(±0.205)	0.284(±0.098)	0.156(±0.135)	N/a
Diverse	0.292(±0.774)	0.922(±0.819)	1.075(0.876)	1.88(±1.297)	N/a	N/a	N/a	N/a	**13.571(±12.908)**
(DUD-E)	0.005(±0.015)	0.021(0.016)	0.023(±0.018)	0.031(±0.022)	**0.295(±0.180)**	0.258(±0.170)	0.118(±0.093)	0.104(±0.059)	N/a

In summary, the proposed Perceiver CPI achieves a competitive or better performance than SOTA deep-learning baselines in all settings, due to the fact that our model adopts the strength of a attention mechanism to dynamically adjust the features of drugs and proteins in different combinations.

### 3.2 Discussion

#### 3.2.1 Difference between perceiver CPI and perceiver IO

**Fig. 3.**
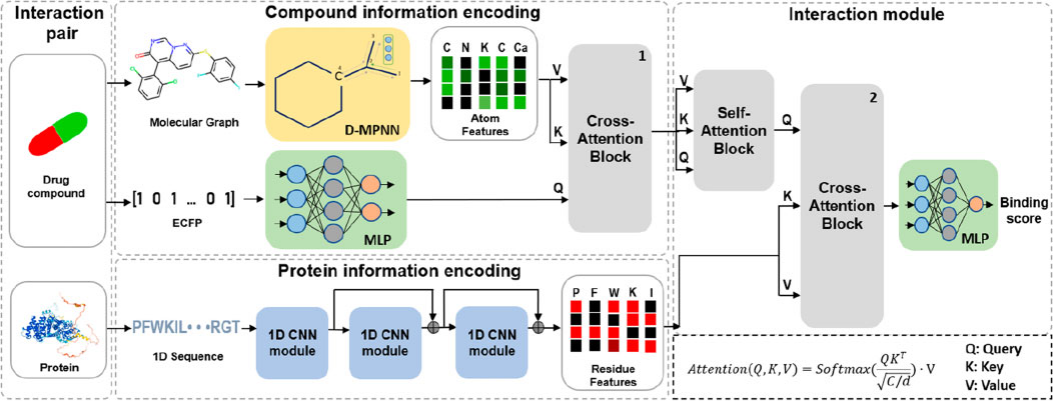
Overview of perceiver CPI. The model is a combination of three-element neural networks that take information from three sources: molecular graphs, Morgan fingerprints for constructing compound patterns and protein sequences for extracting protein knowledge

Perceiver IO is an updated version of Perceiver, which uses an asymmetric attention mechanism to accept input information into a tight latent space. Subsequently, the output of Perceiver is merged with the query system using an additional cross-attention. The key insight is to produce each output by attending to the latent array using a specific output query associated with that output. Therefore, the target of Perceiver IO is the input compound. However, the purpose of Perceiver CPI is to seek a change in the protein caused by the effect of a compound; hence, our target is the input protein. As shown in Figure 3, we take the key (K) and value (V) from the protein information, contrary to Perceiver IO, which considers the protein information as an output query array (Q). Besides, empirical experiment results with the original structure of Perceiver IO showed poorer performance on the CPI task when compared to Perceiver CPI.

## 4 Conclusion and future work

In this study, we deployed cross-attention mechanisms to address the CPI task. We proposed a novel attention mechanism to not only enrich the information extracted from a compound using ECFP knowledge but also to capture CPI information effectively. The proposed Perceiver CPI model exhibited a significantly improved performance on three benchmark datasets when compared with SOTA baselines in terms of MSE and CI.

Although Perceiver CPI has demonstrated excellent performance, much work remains to improve the performance of CPI prediction tasks in the future.

- Finding and extracting meaningful features from proteins remains a difficult but worthwhile task. For instance, AlphaFold2 from DeepMind can be used to predict the 3D structure of proteins (Jumper *et al.*, 2021).
- The information taken from compounds can still be cultivated more profitably, such as by using the META-Learning method to construct a better representation from small datasets.
- Utilizing information from 3D structures produced from SMILES, as GeoMol attempts to do, is also a promising method because of its high information capacity (Ganea *et al.*, 2021).
- Adopting the transfer learning method for individual neural networks (compound and protein networks) to generate improved representations from the beginning with the help of prior knowledge should also be considered.
- The interpretability of Perceiver CPI is limited by the dimensionality reduction of MLP from the hidden state update process in the message-passing step and from the attention blocks. Addressing such useful features would form a valuable part of future work.

## Supplementary Material

btac731_Supplementary_Data

## Data Availability

Our study used open-access datasets, and the data-related links are available in the Data availability section in the supplementary document.
