# Supplementary material for: Perceiver CPI: a nested cross-attention network for compound–protein interaction prediction
Source: Bioinformatics. 2022 Nov 23;39(1):btac731. doi: 10.1093/bioinformatics/btac731 (PMC9848062; doi:10.1093/bioinformatics/btac731)
Supplement: btac731_Supplementary_Data [file btac731_supplementary_data.pdf]

Supplemental Information for: "Perceiver CPI: A nested cross-attention network for compound-protein interaction prediction"

Ngoc-Quang Nguyen[1], Gwanghoon Jang[1], Hajung Kim[2], and Jaewoo Kang[1,2,3,*]

[1]Department of Computer Science and Engineering, Korea University, Seoul, Republic of Korea

[2]Interdisciplinary Graduate Program in Bioinformatics, Korea University, Seoul, Republic of Korea

[3]AIGEN Sciences, Seoul, 04778, Republic of Korea

[*]Corresponding author: kangj@korea.ac.kr

1 Supplementary

1.1 Message passing mechanism

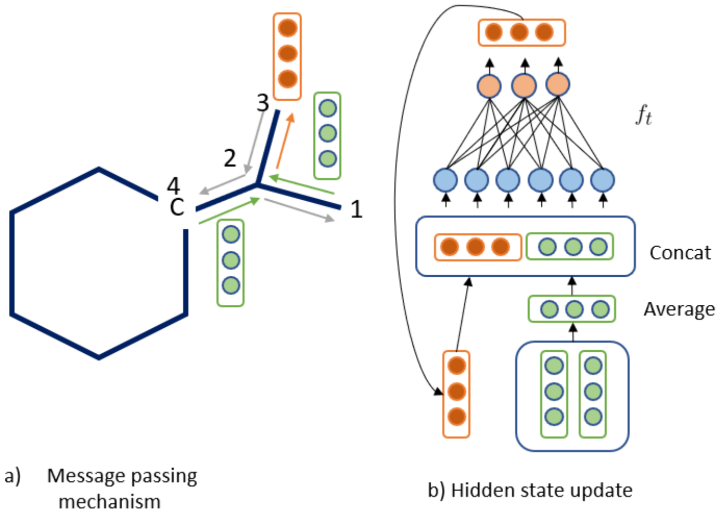

Figure 1: The message passing mechanism of DMPNN (Yang et al., 2019). a) The message from 2->3 is only propagated to node 4 and after. The message contains both information from atom and bond such as: atom type, atomic mass, in ring or not etc... b) Hidden state update mechanism.

1.2 Weighted loss function

Imbalanced datasets have posed a challenge for predictive modeling. They result in models with poor predictive performance, specifically for minority data points. To address this issue, we apply the weighted loss function.

$$\begin{cases} L_{(y,\hat{y})_1} = \alpha \times \frac{1}{N} \sum_{i=0}^{N} (y - \hat{y}_i)^2, if(y \in [\tau_1, \tau_2]) \\ L_{(y,\hat{y})_2} = \beta \times \frac{1}{N} \sum_{i=0}^{N} (y - \hat{y}_i)^2, if(y \notin [\tau_1, \tau_2]) \\ L = L_{(y,\hat{y})_1} + L_{(y,\hat{y})_2} \end{cases} \tag{1}$$

where N is the number of instances and $[\tau_1, \tau_2]$ is the criterion. α and β are the weighted constants. Notably, β is greater than α; using this technique, we intentionally force the model to perform a larger weight update for data points that are not in the range $[\tau_1, \tau_2]$, whereas the other has less impact on the update. We empirical applied the loss funtion to Davis and KIBA dataset due to their typical uneven distribution. Owning to the experimental results, we chose α as 0.5 and β as 5. While $[\tau_1, \tau_2]$ is picked following the density estimation of experimental datasets. To be more specific, $[\tau_1, \tau_2]$ is $[0, 5]$, and is $[11.1, 12]$ on Davis dataset and KIBA dataset, respectively.

1.3 Additional experiments

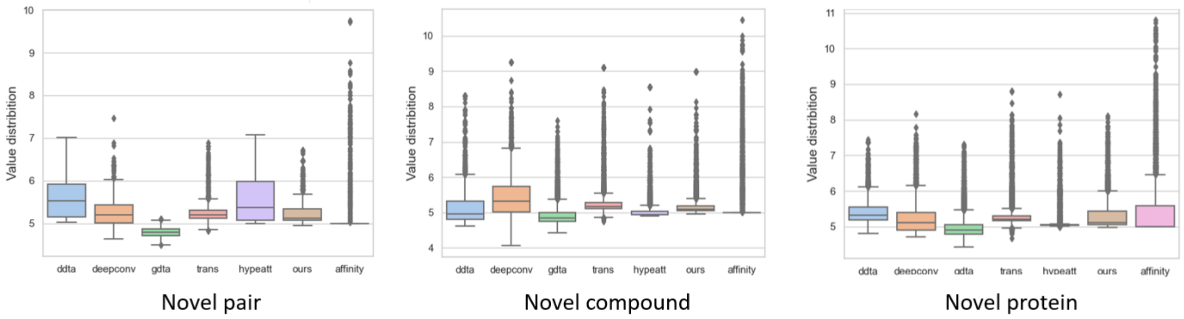

Figure 2: The visualization of prediction for first folds of each setting from the Davis dataset. From left to right: DeepDTA (Öztürk et al., 2018), DeepConvDTI (Lee et al., 2019), GraphDTA (Nguyen et al., 2021), TransformerCPI (Chen et al., 2020), HyperattentionDTI (Zhao et al., 2022), Perceiver CPI (ours), and the label. Here, we confirmed that the prediction distribution of Perceiver CPI is closely mimic to the distribution from the label. Which revealed that our method successfully capture the information that is related to the compound-protein interaction. As can be seen from the distribution, some of the baselines have predictions that are smaller than five, but, there is no such value in Davis dataset. Nevertheless, our model keeps predicting the binding affinity greater or equal to five.

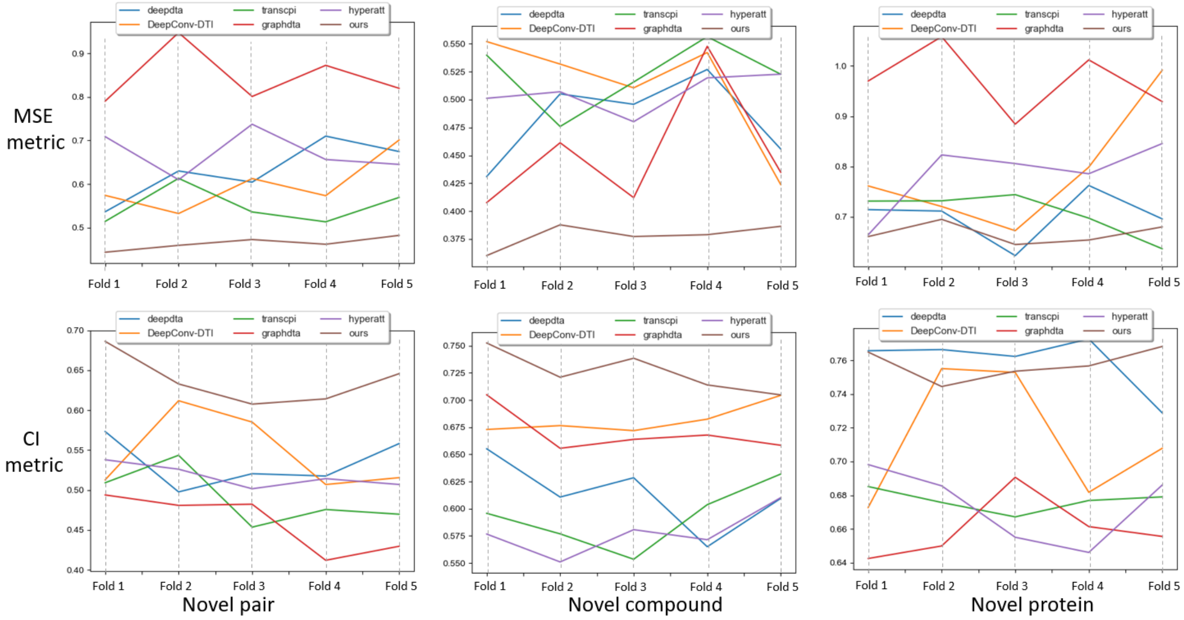

Figure 3: The visualization of MSE (the lower, the better) and CI (the higher, the better) for five-fold of three settings on the Davis dataset. The performance of Perceiver CPI on novel pair and novel compound settings totally outperformed SOTA baseline models, while it showed the competitive achievement with competitors on a novel protein setting.

1.4 Result of Perceiver CPI on classification problem

Table 1: Statistic of GPCR dataset.

Proteins	Compounds	Positive pairs	Negative pairs	Density (%)
356	5359	7989	7354	0.8

Table 2: Classification result for the comparison between Perceiver CPI and classifier competitors for novel pair settings on GPCR dataset.

Model	AUC	Precision	Recall	Accuracy
DeepConvDTI	0.451(±0.116)	0.481(±0.180)	0.464(±0.068)	0.460 (±0.089)
TransformerCPI	0.500(±0.038)	0.519(±0.105)	0.496(±0.023)	0.510(±0.052)
HyperattentionDTI	0.511(±0.050)	0.522(±0.126)	0.505(±0.026)	0.525(±0.043)
PerceiverCPI	**0.605(±0.110)**	**0.607(±0.178)**	**0.544(±0.047)**	**0.590(±0.070)**

Table 3: Statistic of GPCR Subset (5 Targets) and Diverse Subset (7 Targets) from DUD-E database.

Targets (Gpcr Subset)	Actives	Decoys
AA2AR (Adenosine A2a receptor (GPCR))	482	31,550
ADRB1 (Beta-1 adrenergic receptor (GPCR))	247	15,850
ADRB2 (Beta-2 adrenergic receptor (GPCR))	231	15,000
CXCR4 (C-X-C chemokine receptor type 4 (GPCR))	40	3,406
DRD3 (Dopamine D3 receptor (GPCR))	480	34,050
**Targets (Diverse Subset)**	**Actives**	**Decoys**
AKT1 (Serine/threonine-protein kinase AKT)	293	16,450
AMPC (Beta-lactamase)	48	2,850
CP3A4 (Cytochrome P450 3A4)	170	11,800
GCR (Glucocorticoid receptor)	258	15,000
HIVPR (Human immunodeficiency virus type 1 protease)	536	35,750
HIVRT (Human immunodeficiency virus type 1 reverse transcriptase)	338	18,891
KIF11 (Kinesin-like protein 1)	116	6,850

1.5 Ablation study

To verify the efficiency of each contribution in the Perceiver CPI architecture, we performed an ablation test in which we removed each module individually inside the network. The results highlighted the importance of the entire network in all three tests (the first fold for each setting). To analyze the performance of each network fairly, the quantity of datasets for the training and test ratios also remained unchanged, as in the previous experiments. For instance, we detached the D-MPNN in Model 0, which only contained ECFP, 1DCNN, and CABs, before moving to the MLP layers. The results in Table 4 demonstrate that CABs played an essential role in extracting CPI features in interaction pairs, improving performance on all three tasks. Strikingly, without the appearance of CAB-2, the performance of Model 4 was slightly better than that of Model 3, where CAB-1 was removed. Specifically, when experimenting with the importance of CAB in Models 3, 4, and 5, we replaced CAB with simple concatenations. When both of CABs were removed from Model 5, its prediction ability decreased remarkably. Overall, losing any one of the contributions to the framework degraded the model quality.

Table 4: Ablation study of Perceiver CPI on first folds of three settings from Davis dataset (SAB: self-attention block, CAB: cross-attention block).

Model	D-MPNN	ECFP	SAB	CAB (1)	CAB (2)	MSE/CI (novel pair novel compound novel protein)
Model 0		✓	✓	✓	✓	0.486/0.567<br>0.420/0.674<br>0.673/0.687
Model 1	✓		✓	✓	✓	0.496/0.573<br>0.403/0.684<br>0.710/0.748
Model 2	✓	✓		✓	✓	0.510/0.640<br>0.437/0.707<br>0.728/0.748
Model 3	✓	✓	✓		✓	0.475/0.600<br>0.443/0.656<br>0.750/0.720
Model 4	✓	✓	✓	✓		0.461/0.612<br>0.391/0.722<br>0.742/0.736
Model 5	✓	✓	✓			0.844/0.543<br>0.424/0.678<br>0.744/0.731
Perceiver CPI	✓	✓	✓	✓	✓	**0.442/0.685**<br>**0.358/0.751**<br>**0.660/0.755**

Table 5: The Atom's and Bond's feature ablation study.

Model	Atom num	Formal charge	Chiral tag	Hs	Hydrization	Bond feature	MSE/CI (novel pair novel compound novel protein)	MSE/CI Change (Compared to Perceiver CPI)
Model 0		✓	✓	✓	✓	✓	0.536/0.540<br>0.419/0.653<br>0.749/0.715	0.094/-0.145<br>0.061/-0.098<br>0.089/-0.04
Model 1	✓		✓	✓	✓	✓	0.475/0.608<br>0.382/0.703<br>0.660/0.757	0.033/-0.077<br>0.024/-0.048<br>0/0.002
Model 2	✓	✓		✓	✓	✓	0.475/0.685<br>0.358/0.750<br>0.663/0.753	0.033/0<br>0/-0.001<br>0.003/-0.002
Model 3	✓	✓	✓		✓	✓	0.531/0.552<br>0.360/0.740<br>0.667/0.758	0.089/-0.133<br>0.002/-0.011<br>0.007/0.003
Model 4	✓	✓	✓	✓		✓	0.477/0.638<br>0.355/0.744<br>0.660/0.759	0.035/-0.047<br>-0.003/-0.007<br>0/0.004
Model 5	✓	✓	✓	✓	✓		0.521/0.571<br>0.414/0.693<br>0.674/0.760	0.079/-0.114<br>0.056/-0.058<br>0.014/0.005
Perceiver CPI	✓	✓	✓	✓	✓	✓	0.442/0.685<br>0.358/0.751<br>0.660/0.755	

In The Atom's and Bond's feature ablation study, we independently turn off testing features by turning them into 0. Note that in our model we used the same method proposed by original D-MPNN, in which all of the features were initialized by the open source toolkit for cheminformatics RDKit. The Table 5 shows the performance of Perceiver CPI while missing atom features. The performance of the model drastically decreases when the Atom num features (atom type) is not used. Nevertheless, when the Formal charge feature is not used, the performance of the model is slightly increased on novel protein task.

1.6 Similarity check

In protein similarity calculation, the number of amino acids in alignment divided by the length of the protein sequence (we used 500 for Perceiver CPI) as shown in the following equation :

$$Similarity_{prot} = \frac{\text{number of aligned amino acids}}{\text{total length of the sequence}} \tag{2}$$

Table 6: Similarity of training set and testing set in terms of Tanimoto similarity (for compounds), Protein similarity (for proteins).

Experiments	Tanimoto Similarity	Protein Similarity	Min/Max Similarity (Compound)	Min/Max Similarity (Protein)
Davis	0.135(±0.004)	0.071(±0.009)	0.048/0.697	0.047/0.856
KIBA	0.117(±0.002)	0.071(±0.0001)	0.009/0.857	0.049/0.623
Metz	0.128(±0.003)	0.071(±0.0002)	0.052/0.591	0/1
Cross Domain	0.068(±0.012)	0.098(±0.037)	0.041/1	0/1
DUD-E (GPCR-GPCR)	0.111(±0.034)	0.092(±0.031)	0.0/0.895	0.052/0.692
DUD-E (GPCR-Diverse)	0.111(±0.035)	0.069(±0.013)	0.0/0.894	0.044/0.155

Table 7: Detailed enrichment factor analysis results for GPCR and Diverse subsets from the DUD-E database.($EF_{1\%}$ / $BEDROC_{\alpha=80.5}$).

Target	Family	DeepConvDTI	TransformerCPI	HyperattentionDTI	Perceiver CPI (ours)	Gold	Glide	Surflex	FlexX	Blaster
AA2AR	GPRCR	12.95/0.18	2.83/0.09	2.02/0.06	8.23/0.15	-/**0.29**	-/0.13	-/0.34	-/0.17	**22**/-
ADRB1	GPRCR	4.76/0.10	0.41/0.00	0.00/0.00	9.12/0.17	-/**0.43**	-/0.31	-/0.25	-/0.18	**11**/-
ADRB2	GPRCR	0.00/0.00	0.00/0.00	4.92/0.09	**18.23**/0.30	-/0.43	-/**0.50**	-/0.41	-/0.36	4/-
CXCR4	GPRCR	2.49/0.04	0.83/0.00	4.78/0.05	3.12/0.05	-/0.08	-/0.01	-/**0.27**	-/0.01	**18**/-
DRD3	GPRCR	28.44/0.44	0.0/0.00	8.19/0.16	**43.13**/**0.51**	-/0.18	-/0.04	-/0.15	-/0.06	**4**/-
AMPC	Miscellaneous	0.00/0.00	0.00/0.00	0.00/0.00	0.00/0.00	-/0.04	-/**0.09**	-/0.00	-/0.04	**8**/-
CP3A4	CYP450	0.00/0.00	1.17/0.03	2.35/0.03	**4.11**/0.07	-/**0.21**	-/0.17	-/0.13	-/0.08	2/-
HIVPR	Miscellaneous	2.05/0.04	0.37/0.01	1.30/0.02	2.42/0.04	-/**0.30**	-/0.14	-/0.10	-/0.05	**5**/-
HIVRT	Miscellaneous	0.00/0.00	2.06/0.03	0.29/0.01	1.76/0.03	-/**0.42**	-/0.37	-/0.13	-/0.19	**7**/-
KIF11	Miscellaneous	0.00/0.00	1.71/0.04	1.71/0.04	0.85/0.01	-/0.55	-/**0.59**	-/0.12	-/0.08	**35**/-
AKT1	Protein kinases	0.00/0.00	0.00/0.00	0.34/0.01	1.70/0.04	-/**0.42**	-/0.24	-/0.05	-/0.11	**29**/-
GCR	Nuclear receptors	0.00/0.00	1.15/0.03	1.54/0.05	2.32/0.03	-/0.13	-/0.21	-/**0.30**	-/0.18	**9**/-

2 Data availability

The related links are as follows:
KIBA, Davis: https://github.com/kexinhuang12345/DeepPurpose
Metz: https://github.com/sirimullalab/KinasepKipred
PDBBind: https://github.com/lishuya17/MONN
GPCR (train):https://github.com/lifanchen-simm/transformerCPI
DUD-E GPCR (test):http://dude.docking.org/subsets/gpcr
DUD-E Diverse (test):http://dude.docking.org/subsets/diverse

References

Chen, L., Tan, X., Wang, D., Zhong, F., Liu, X., Yang, T., ... Zheng, M. (2020). Transformercpi: improving compound–protein interaction prediction by sequence-based deep learning with self-attention mechanism and label reversal experiments. *Bioinformatics*, *36*(16), 4406–4414.

Lee, I., Keum, J., & Nam, H. (2019). Deepconv-dti: Prediction of drug-target interactions via deep learning with convolution on protein sequences. *PLoS computational biology*, *15*(6), e1007129.

Nguyen, T., Le, H., Quinn, T. P., Nguyen, T., Le, T. D., & Venkatesh, S. (2021). Graphdta: Predicting drug–target binding affinity with graph neural networks. *Bioinformatics*, *37*(8), 1140–1147.

Öztürk, H., Özgür, A., & Ozkirimli, E. (2018). Deepdta: deep drug–target binding affinity prediction. *Bioinformatics*, *34*(17), i821–i829.

Yang, K., Swanson, K., Jin, W., Coley, C., Eiden, P., Gao, H., ... others (2019). Analyzing learned molecular representations for property prediction. *Journal of chemical information and modeling*, *59*(8), 3370–3388.

Zhao, Q., Zhao, H., Zheng, K., & Wang, J. (2022). Hyperattentiondti: improving drug–protein interaction prediction by sequence-based deep learning with attention mechanism. *Bioinformatics*, *38*(3), 655–662.
